# Exercise-Based Injury Prevention in High-Level and Professional Athletes: Narrative Review and Proposed Standard Operating Procedure for Future Lockdown-Like Contexts After COVID-19

**DOI:** 10.3389/fspor.2021.745765

**Published:** 2021-12-17

**Authors:** Géraldine Martens, François Delvaux, Bénédicte Forthomme, Jean-François Kaux, Axel Urhausen, François Bieuzen, Suzanne Leclerc, Laurent Winkler, Franck Brocherie, Mathieu Nedelec, Antonio J. Morales-Artacho, Alexis Ruffault, Anne-Claire Macquet, Gaël Guilhem, Didier Hannouche, Philippe M. Tscholl, Romain Seil, Pascal Edouard, Jean-Louis Croisier

**Affiliations:** ^1^Réseau Francophone Olympique de la Recherche en Médecine du Sport (ReFORM) International Olympic Committee (IOC) Research Centre for Prevention of Injury and Protection of Athlete Health, Liège, Belgium; ^2^Physical Medicine and Sport Traumatology Department, SportS^2^, FIFA Medical Centre of Excellence, Fédération Internationale de Médecine du Sport (FIMS) Collaborative Centre of Sports Medicine, University of Liège and University Hospital of Liège, Liège, Belgium; ^3^Laboratory of Human Motion Analysis, University of Liege, Liège, Belgium; ^4^Luxembourg Institute of Research in Orthopedics, Sports Medicine and Science, Luxembourg, Luxembourg; ^5^Clinique du Sport, Centre Hospitalier de Luxembourg, Luxembourg, Luxembourg; ^6^Human Motion, Orthopedics, Sports Medicine and Digital Methods, Luxembourg Institute of Health, Luxembourg, Luxembourg; ^7^Medico-Scientific Department, Institut National du Sport du Québec (INS), Montréal, QC, Canada; ^8^French Institute of Sport (INSEP), Paris, France; ^9^Laboratory Sport, Expertise and Performance (EA 7370), French Institute of Sport (INSEP), Paris, France; ^10^Unité de Recherche Interfacultaire Santé et Société (URiSS), Université de Liège, Liège, Belgium; ^11^Department of Orthopaedic Surgery and Traumatology, Geneva University Hospitals, Geneva, Switzerland; ^12^Service de Chirurgie Orthopédique, Centre Hospitalier de Luxembourg, Luxembourg, Luxembourg; ^13^Department of Clinical and Exercise Physiology, Sports Medicine Unit, University Hospital of Saint-Etienne, Saint-Etienne, France; ^14^Inter-University Laboratory of Human Movement Science (LIBM EA 7424), University of Lyon, University Jean Monnet, Saint Etienne, France

**Keywords:** sports injury prevention, team work, communication, injury risk, athlete's health

## Abstract

In regular times, implementing exercise-based injury prevention programs into the training routine of high-level and professional athletes represents a key and challenging aspect to decrease injury risk. Barriers to implementing such prevention programs have previously been identified such as lack of resources, logistic issues or motivation. The COVID-19 pandemic associated with restrictions on daily life dramatically impacted sports participation from training to competition. It is therefore reasonable to assume that such lockdown-like context has exacerbated the challenge to implement exercise-based injury prevention programs, potentially leading to a greater musculoskeletal injury risk. In this narrative review, recommendations are proposed for building an expertise- and evidence-based Standard Operating Procedure for injury prevention in lockdown-like contexts for high-level and professional athletes. The following recommendations can be provided: (1) assess the global and sport-specific risks in the light of the ongoing cause of isolation; (2) adapt remote training materials and programs; (3) ensure regular quality communication within the staff, between athletes and the staff as well as between athletes; (4) follow the athlete's mental well-being; and (5) plan for a safe return-to-sports as well as for an ongoing monitoring of the load-recovery balance. These key domains should further be addressed to comply with local policies, which are subject to change over time in each individual country. The use of these recommendations may improve the readiness of athletes, coaches, physicians and all sports stakeholders for future lockdown-like contexts.

## Introduction

Sports injury prevention has become an essential field of sports medicine and science. It aims at reducing the impact of injuries on sports participation and improving long-term health (Engebretsen and Bahr, 2005). Implementing exercise-based prevention programs has been shown to be efficient in reducing the occurrence of injuries (Lauersen et al., 2014) but is still poorly applied on the field (Bahr et al., 2015). The major COVID-19 pandemic might have further compromised this implementation by drastically restricting daily life and physical activities. As most communities are preparing to live with the SARS-CoV-2 virus for a long haul, and since successive lockdown-like situations are likely to happen in the future either from this virus, other pandemics or other similar contexts, there is a need to anticipate and establish future recommendations to help exercise-based prevention program implementation in field contexts. The aim of this work was therefore two-fold: (1) to review the current literature on the impact of COVID-19 on sports participation and identify potential gaps in terms of injury prevention and (2) to provide Standard Operating Procedure to promote and conduct exercise-based injury prevention in high-level or professional athletes under lockdown-like contexts, based on both the available literature and the international sports medicine and science experiences.

## How May the COVID-19 Pandemic Have Affected Injury Prevention Programs?

Sports-related injuries can have severe consequences affecting physical, psychological and social aspects, especially in high-level and professional athletes (Van Mechelen, 1997). Injury prevention approaches are therefore crucial to protect the athlete's health (Engebretsen and Bahr, 2005). Based on previous theoretical frameworks (van Mechelen et al., 1992; Meeuwisse, 1994; Finch, 2006; Bittencourt et al., 2016), robust evidence on how exercise-based injury prevention approaches effectively decrease the occurrence of injuries in both amateur and high-level or professional athletes has been growing (Lauersen et al., 2014). Introducing standardized neuromuscular training programs (such as FIFA 11+, OSTRC Shoulder Injury Prevention Programme, Nordic hamstring exercise) significantly reduces the rate of injuries in elite-level handball (Myklebust et al., 2003; Andersson et al., 2017), soccer (Arnason et al., 2008; Petersen et al., 2011) or basketball (Longo et al., 2012) teams. Higher compliance leads to lower injury rates, thus indicating that these protective effects appear to be dose-dependent (Soligard et al., 2010; Hägglund et al., 2013; Silvers-Granelli et al., 2018). However, several studies showed poor implementation and adherence of prevention programs in soccer, despite these well-known benefits (Bahr et al., 2015; O'Brien and Finch, 2016; van der Horst et al., 2018; Slauterbeck et al., 2019). Coaches and staff members identified a series of explanatory factors such as peer social support; limited access to resources; athletes' perception; lack of team work and communication; lack of knowledge of the programs and under staffing (O'Brien and Finch, 2016; Lindblom et al., 2018). Athletes themselves reported personal motivation and knowledge of the program as key factors for compliance (van der Horst et al., 2018). Additional deleterious factors (e.g., training cancellation, loss of access to field and materials or forced self-managed training) may therefore negatively impact the efforts directed toward injury prevention, potentially leading to serious consequences.

The outbreak of the SARS-CoV-2 causing the COVID-19 pandemic is one of the largest public health emergency states of our time, affecting every aspect of life as it was known before. The most striking example for sports being the postponement of the 2020 Tokyo Olympic Games which was last time seen during World War II. The training (except for a handful of elite athletes who benefited from a “Quarantine Training Camp”) (Washif et al., 2021) and competition routines have been tremendously disrupted by the lockdown policies, which were embraced by most governments to contain the spread of the deadly COVID-19 (WHO, 2020a,b). With regards to injury prevention programs specifically, numerous factors consequently affected their potential use.

First of all, the difficulty to access specialized equipment might have complicated both injury screening tests and prevention exercises. Although debated (Bahr, 2016; Hewett, 2016; Verhagen et al., 2018), regular laboratory and field evaluations of neuromuscular control, function, fitness, coordination, flexibility, strength levels and (im)balances do not only allow to identify an individual injury risk profile (Hewett et al., 2005; Croisier et al., 2008; Rommers et al., 2020), but also to build individualized prevention programs addressing each athlete's needs (e.g., neuromuscular training, selective hamstring eccentric strengthening, stretching) (Roe et al., 2017; Welch et al., 2020). Without this kind of information, athletes and coaches can have difficulties to objectively assess individual deficiencies that can force to blindly apply “one-size-fits-all” injury prevention training, lacking specificity. Then, applying these prevention programs also often requires specific equipment and infrastructure such as ergometers, bars, balls, practice fields, gyms or strength training rooms for which access might also have been compromised—even though recent recommendations specifically addressed the possible adaptations of training infrastructures (Gentil et al., 2020). Likewise, many athletes were not able to add sport-specific movement patterns to their neuromuscular injury prevention training program and lacked the necessary ecological conditions (Hewett et al., 2005; Finch, 2006).

Second, even in today's highly digitalized environment, compliance with any type of injury prevention program may be hindered without in-person support from the coaches and the training partners (van der Horst et al., 2018) and in absence of the usual structured daily routine. As introduced above, several barriers such as the monotonous nature of the exercises, the lack of objective measures as well as the lack of structure and support have also been identified (O'Brien and Finch, 2016; van der Horst et al., 2018) and were potentially magnified during home-based or restricted training without peers. Specific examples on how to tackle these issues have however been provided by some sports organizations who set up group virtual trainings with gradual challenges to maintain motivation and engagement.

Third, beyond exercise-based injury prevention training programs, the pandemic has psychologically impacted athletes, with considerable individual differences, as confirmed by recent data (Reardon et al., 2021). In (semi-)elite South African athletes, for instance, 52% of 692 surveyed athletes reported feeling depressed during the lockdown period, with a significantly higher rate in female athletes (Pillay et al., 2020). Individual sports athletes were also prone to higher levels of psychological distress as compared to team sports athletes, as shown in a similar study performed with 64 professional and non-professional Nigerian athletes (Uroh and Adewunmi, 2021). Another prospective study followed a cohort of 15 high-level Australian athletes over 18 weeks and showed higher risk of adjustment disorder (i.e., failure to cope with a stressor expressed by depressive symptoms) following lockdown measures as compared to pre-pandemic baseline levels (Simons et al., 2021). Many stressors were reported including the toll of isolation, the lack of access to training facilities, the uncertainty about the future and the cancellation of competitive events. These factors on top of the regular change of obligations and guidelines from authorities, the fear of contagion (oneself or relatives), the lack of short-term objectives and the subsequent loss of motivation had their own impact on prevention exercises (for which enthusiasm can be at stake given the low reported compliance). As a matter of fact, many other injury risk factors may have been affected: sleep, nutrition, lifestyle, mental training, medical follow-up, etc. Even though the present work does not address the ambitious aim of accounting for all these variables, it should be kept in mind as the nature of injury prevention is multifactorial in essence (Bittencourt et al., 2016).

Finally, the return on the field and the daily routine athletes were used to before the pandemic has been challenging from many perspectives. The enforced restricted training period prolonged itself well beyond the regular off-season breaks, where a fast decline of performance variables (endurance- and neuromuscular-related) is observed (Mujika and Padilla, 2000b; Silva et al., 2016). Several recent reports about training maintenance during lockdown or restraining measures show a non-negligible decrease in training activity, concerning duration, intensity and sport-specific movement patterns (Muriel et al., 2020; Zinner et al., 2020). In international professional cycling, for instance, a 7-week home confinement period led to a significant decrease in both training volume (about 34%) and power output performances (between 1 and 19%), even with home-based individual training, in a team of 18 cyclists (Muriel et al., 2020). In high-level kayak and canoe, a retrospective comparison of wearable sensors data 4 weeks prior and 4 weeks following the local lockdown measures (Germany) revealed a reduced training time (−27.6%), shorter sessions (−15.1%) and more training time below 60% of the individual peak heart rate (+9.7%) (Zinner et al., 2020). This poses a non-negligible injury risk as detraining rapidly negatively impacts muscle-tendon properties (Kubo et al., 2010) and cardiorespiratory adaptations (decline in maximal oxygen uptake, blood volume, maximal cardiac output, ventilator efficiency and endurance performance) (Mujika and Padilla, 2000a,b). Confirming these deleterious effects, recent longitudinal data in a small sample of 16 elite soccer players showed significant deterioration of neuromuscular aspects (preparation for eccentric deceleration during jumping and landing) of the routine countermovement jump tests after 15 weeks of isolated training (Cohen et al., 2020). This decrease was not observed following the players' regular off-season breaks of 23 days as these variables remained stable, emphasizing the impact of prolonged off-pitch training (Cohen et al., 2020).

At return to sport, athletes also had to face a workload increase (in duration, frequency and intensity), with multiple rescheduled (and potentially overlapping) competitions over a congested fixture period, which is known to increase the workload (potentially leading to an Overtraining Syndrome) (Meeusen et al., 2006) as well as the rate of injuries (Dupont et al., 2010; Bengtsson et al., 2018; Mohr et al., 2020). Depending on the training conditions during lockdown and its duration, a greater amount of time might be necessary to come back at the baseline fitness level and thereby reduce the risk of injury (Brocherie et al., 2020). In elite football for instance, if this period lasts for about 4 weeks with more than 50% decrease in workload, the recommended duration for returning to competitive activities is about 3–5 weeks (Mohr et al., 2020). Rapid detraining (8 weeks) and considerably longer retraining up to previous fitness level (20 weeks) has also been demonstrated for rowing (Godfrey et al., 2005). A smaller-scale example of unplanned reduced training followed by premature return to competition is provided by the 2011 National Football League Lockout that lasted more than 4 months. Obviously, players had a drastic reduction (about 2 weeks instead of 9 – and previously 14 –) of their preseason training which resulted in a dramatic increase (+400%) in Achilles tendon rupture prevalence over the course of a few weeks during preseason (Myer et al., 2011). The available recent data from the German Bundesliga soccer league confirms this scenario: there was a drastic increase (+226%) in the global injury rate (from 0.27 to 0.84 injuries per game on the week post-lockdown after a 66-day break) following the early return to competition (Mason, 2020; Seshadri et al., 2021). This further calls for an optimized preparation to the return to high-level and professional sports.

These drawbacks might be partially balanced by some potential advantages of this prolonged period without organized competitions. Athletes had more time for recovery, sleep or mental training for instance; potentially preventing Overtraining Syndrome (Meeusen et al., 2006). Some athletes who were already injured could have benefited from a resting period without compromising their future participation to later major competitions. Others might have taken advantage of the lightened agenda to focus on known deficits or on exercises they lacked time to perform and adjust their prevention training accordingly (Jukic et al., 2020). These speculations will have to be answered by ongoing research initiatives but suggest these peculiar lockdown-like periods could be an important window of opportunity for injury prevention. The lessons learnt from this unprecedented situation can shape future practice by being better prepared, as similar important events might happen in the near future (e.g., consecutive pandemic waves, other sanitary or social crisis). This would however need a structured operational plan.

## The Need for an Operational Plan in Case of Lockdown-Like Contexts

**Figure 1:**
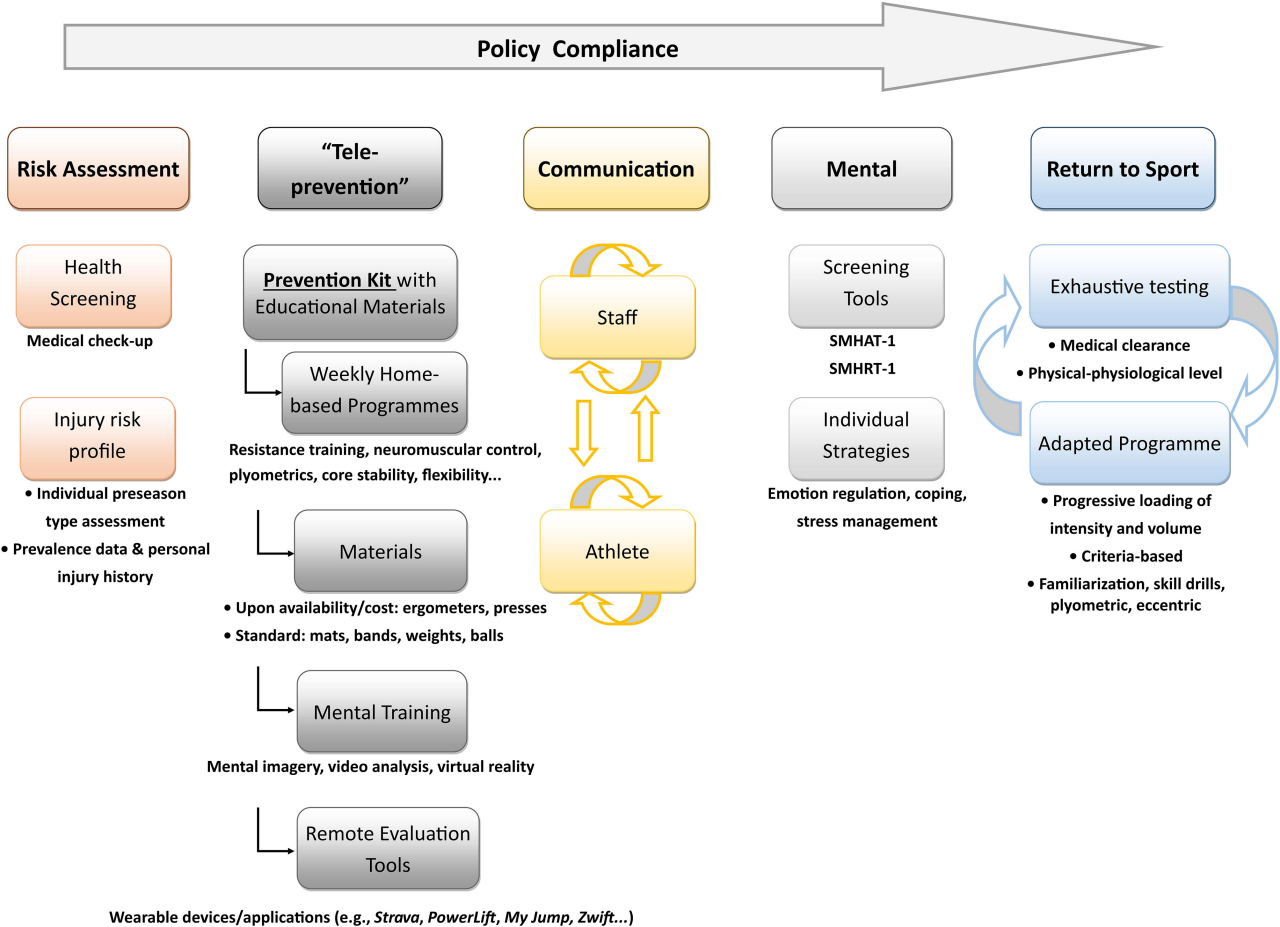
Checklist of tools and procedures for injury prevention that should be readily available before a lockdown-like situation.

While the precise consequences of this pandemic on sports-related injuries are still under investigation, sports injury prevention stakeholders should now be ready to switch to a remote training program at any given time. Organized sports structures should have Standard Operating Procedures ready based on the checklist provided in Figure 1 and addressing these guidelines in their specific environment with the associated potential logistic and economic barriers. We suggest that these procedures should be based on the following:

### Global Health Risk Assessment

Preserving the individual health by avoiding exposure to any hazard has the highest priority. For any cause of lockdown or restriction orders (viral pandemic, pollution peaks, social crisis…), the specific risks related to the situation forcing athletes to isolate have to be assessed regularly as these situations can be rapidly evolving. If the cause of lockdown-type measures is a viral pandemic, athletes should ideally be properly screened even in the absence of symptoms to prevent these increased risks in the community (Dores and Cardim, 2020; Toresdahl and Asif, 2020) and isolated when required (Rankin and Heron, 2020). In the specific case of the COVID-19, guidelines for infected athletes' medical evaluation recommend adapted check-up, based on algorithms depending on individual risk stratification, including blood sampling and electrocardiogram in order to screen for cardiorespiratory complications as well as other organ failures (Fabre et al., 2020; Nieß et al., 2020; Wilson et al., 2020; Kim et al., 2021). Even if hospitalization rates appear to be lower in individuals with higher maximal exercise capacity (Brawner et al., 2020), this virus can affect the cardiovascular system of athletes leading to a non-negligible risk of myocarditis in addition to the respiratory impairments commonly described (Guzik et al., 2020; Huang et al., 2020; Kim et al., 2021). As a matter of fact, these potential complications have to be detected early on and resolved before returning to sports.

### Sport-Specific Risk Assessment and ≪Tele-Prevention≫

Regarding the identification of individual injury risk factors, a battery of tests including biomechanical, aerobic, strength and neuromuscular assessments is suggested and often performed during preseason evaluations (Hewett et al., 2005; Croisier et al., 2008; Verhagen et al., 2018). In unexpected situations of restrictions, two scenarios can occur: either the athlete did already benefit from this battery in the past and prevention can thereby be adequately individualized by addressing existing deficits and upon individual progress; or, if this was not the case, solely general prevention programs have to be applied based on commonly encountered risk factors in the practiced sport (van Mechelen et al., 1992). While only the first scenario is ideal and allows capturing the athlete's status at a given time point (before injury or before lockdown), prevention training will have to be performed remotely. This implies:

a. Having ready-to-use weekly exercise-based programs (either individualized or global). These will include muscle strengthening, flexibility, neuromuscular control, balance and agility, core stability, plyometrics, sport-specific movements (of uttermost importance) and endurance in varying degrees according to the sport concerned. The balance between endurance or strength training and sport-specific training will have to be adapted as to compensate the reduced sport-specific training while maintaining overall fitness (Pedersen et al., 2021).
b. Making specific equipment available. Athletes at home should be equipped for cardio- (treadmill, bicycles, rowing ergometers) and resistance training (presses, balls) if possible (Jukic et al., 2020). If not, other frequently-used low cost materials such as mats, skipping ropes, bands or weights should be provided.
c. Incorporating mental training such as mental imagery and video analysis for technical and tactical sport-specific movements. If available, virtual reality tools can represent a significant asset.
d. Using remote-based tools for both evaluation and training purposes. To this end, valid and reliable phone-based applications to measure vertical jump performance or the 1-Repetition maximum, for instance, are already available (Balsalobre-Fernández et al., 2015, 2018; Lim, 2020) and further development of these “tele-screening” approaches should be encouraged. Continuous monitoring of both training load and athlete wellbeing would be further recommended to individually tailor an optimal training. In this remote setting, specific wearable devices and portable technology may be of significant value.

To sum up, since athletes might be highly exposed to the risk of having poor or no access to their usual materials during lockdown, a “prevention survival kit” with specific equipment should be made available, allowing them to perform prevention-oriented exercises at home. Having everything they need in a single kit, along with educational material and professional support, can help incorporating prevention training in athletes' routine practice (Michie et al., 2011).

### Communication: Within the Staff; Between Athlete and Staff; Between Athletes

For high-level and professional athletes' staff, ensuring compliance with remote programs implies a need for continuous communication and feedback. Quality of communication and coach-athlete relationship are associated with positive outcomes, satisfaction and team cohesion (Rhind and Jowett, 2010). In a context of geographical remoteness, a tested and approved communication platform with a designated person for planning and coordinating the virtual meetings should be in place, with several levels of communication:

a. At the staff level, adapted shorter-term remote training plans have to be prepared with the persons in charge of the prevention component (e.g., physician, physiotherapist, coach, physical trainer, sports scientist). Regular internal staff meetings have to be held to set priorities and harmonize interventions around the various training plans and to avoid interferences between different objectives (performance, fitness, prevention). These meetings should emphasize on training contents, priorities, timeframe and adjustment of short-term objectives.
b. At the staff-athlete level, an agreed-upon video-based communication tool will enable fast re-organization and compliance monitoring to the prevention training as well as goal-driven and event-driven adaptations and support. As far as possible, sport-specific exercises should be proposed and motivational steps to enable athletes to commit to the program (e.g., reward feedback, advices) may be an asset. Aside from the coaching, medical follow-up for both health and injury prevention aspects has to be pursued and telemedicine and eHealth solutions should be made available (Dijkstra et al., 2020).
c. At the athletes' level, a dedicated online community platform where athletes can share their feelings and experiences should be made available. Such a platform can limit the effects of isolation and provide opportunities to learn from what they are experiencing. It can also enable athletes to manage their emotions and support one another, as well as comment on what goes well and badly in specific situations and how they adapt or could adapt to achieve positive issues. Such a sharing may enable athletes being creative and inspire others to develop resilience and compliance. Public social media tools can also be used to share training contents, coping strategies and motivational tips, as showed in Boxing for instance (Tjønndal, 2020). Such virtual training tools are indeed associated with higher training frequency and longer duration, as reported in a sample of 329 high-level cyclists (Moreno-Tenas et al., 2021).

### Mental Health Status

As stated above, the COVID-19 related successive lockdowns and/or restraining orders have dramatically impacted a proportion of high-level athletes' mental health (Pillay et al., 2020; Simons et al., 2021; Uroh and Adewunmi, 2021). To address this, physicians, coaches and/or sport psychologists, may measure motivations, emotions, cognitions, metacognitions and behaviors in order to monitor psychological risk factors of injuries and mental disorders during forced isolation (Philippot et al., 2019). The recently developed International Olympic Committee Sport Mental Health Assessment and Recognition Tools will most probably have a key role to play from both healthcare professionals and athletes (and their entourage) perspectives (Gouttebarge et al., 2020). These ready-to-use screening tools for early recognition of mental health symptoms and disorders in the form of several questionnaires have the substantial advantage that they can be remotely administered and thereby immediately made available in lockdown-like situations. Treatment-wise, in light of the COVID-19 pandemic, conventional mental health approaches have to be adapted, as emphasized by recent recommendations (Reardon et al., 2021). Crisis counseling might be privileged for direct effects of the pandemic (morbidity/mortality of loved ones, personal illness). To compensate the loss of bearings, virtual group psychotherapy to partially replicate team dynamics can be considered as well. In order to actually prevent the appearance of mental health symptoms, coaches and/or sport psychologists could provide emotion regulation and coping strategies (e.g., identification, acceptance), as well as techniques to manage acute and chronic stress (e.g., relaxation techniques using breathing exercises, progressive mental exposure to stressors, mindfulness exercises).

### Prepare for Return-To-Sports

As stated above, the enforced lockdowns or restriction measures negatively impacted the athletes' training routine (Muriel et al., 2020; Zinner et al., 2020). This prolonged interruption of regular training and competitions has dramatic detraining consequences on muscle-tendon properties (Kubo et al., 2010), aerobic capacities (Mujika and Padilla, 2000a) and neuromuscular qualities (Cohen et al., 2020; Girardi et al., 2020). It is therefore of paramount importance to assess the detraining effects on an individual basis using a battery of sport-specific tests before planning the retraining program (Girardi et al., 2020; Mohr et al., 2020). Implementing an adapted individualized training before returning to competition could indeed significantly reduce the rate of injuries, in a dose-dependent fashion as shown with European Football Elite (Ekstrand et al., 2020) and a dedicated decision algorithm has been proposed (Brocherie et al., 2020). This specific “back on track” program should consider the training performed during lockdown (including sport-specific mechanical loading) and include a baseline physical and physiological screening, using a shared decision-making approach (Dijkstra et al., 2017; Girardi et al., 2020; Mohr et al., 2020). This program should be continuously updated based on regular medical, physical and physiological assessments while ensuring that the athletes' fitness levels and muscle-tendon mechanical properties get fully restored. The challenge, as compared to routine preseason programs, will be to condense the preseason plan and adapt it to the actual training performed during the prolonged non-competitive period. Recent comprehensive considerations that have been proposed for collision-sports (Stokes et al., 2020) or football (Casais-Martinez et al., 2020; Mohr et al., 2020) can at least partially be extended to other sports: the program should initially focus on gradual refamiliarization of training and competition intensity demands and the focus for the retrained movements (e.g., running, dribbling or passing skills) should be placed on quality first and then on volume. For skill drills, it is preferable to start on smaller spaces and then progressively evolve onto larger fields. Across all domains, the key aspect remains progressivity. There should be a gradual increment of aspects related to intensity (e.g., speed, distance, competitive simulations) and those related to volume (e.g., duration of exercise, number of repetitions) before later introduction of unpredictable variables such as direction changes, decision making or reaction time. High-intensity training activities like plyometric/eccentric loading or sprinting are at higher injury risk. They would therefore need a careful progressive re-introduction to avoid tissue damage (Windt and Gabbett, 2017). Ideally, the program should be criteria-based rather than time-based and in that sense, backwards scheduling the training to meet competitive requirements can be a dangerous path to follow. Lately, a call from both researchers and athletes has been made to account for the physiological detraining aspects, the need for an appropriate pre-season training to decrease injury risk and thereby the need to adapt sports programming accordingly, in light of the unprecedented COVID-19 situation (Sarto et al., 2020). International Sports Federation have indeed a role to play in injury prevention as well, especially in such specific contexts. This is why, in soccer for instance, the FIFA temporarily adapted its playing rules to authorize up to five substitutions per match instead of three (Mota et al., 2021). Similar adaptations could be implemented (e.g., shorter match duration and longer time between consecutive matches) and be made more permanent.

### Compliance With Local Policies

These guidelines should obviously be followed within the framework of the local public health policies that are subject to change from one country to another. The same policies determine the progressive lifting of the lockdown measures, which should be paralleled with the aforementioned return-to-field program, when emphasis is put on collective prevention and identification of individual risk factors while progressively increasing the workload. If possible, communication between representatives of sports organizations and governments to find a common ground which should be focused on preventing the risk of injuries while preserving public health should be encouraged. An appropriate example is provided by the Australian Institute of Sport that developed a specific framework for resuming sport in a COVID-19 environment (Hughes et al., 2020). The policy representatives also have a key role to play with regard to injury prevention and risk mitigation. Indeed, their support in regulating and supporting training opportunities as well as adapting competition agenda is crucial.

Taken together, these guidelines will hopefully shape injury prevention programs in the post-COVID-19 era and/or in comparable lockdown-like situations. The contents *per se* will not be drastically different of the ones conducted before the pandemic but rather the procedures will differ (i.e., individual practice, few materials). In a specific home-based context, particular attention has to be given to athlete's education and compliance. It should be rigorously monitored in a remote setting and fast-evolving technologies may contribute to this aspect. Regular screening assessments and follow-up controls also have an important role to play.

## Conclusion

Among the multiple consequences of the major COVID-19 pandemic, collateral damage may include an increased rate of injuries occurring when returning to sport, with its own socio-economic burden. As anticipation is key, lessons should now be learnt through objective assessment of the impact of this unprecedented crisis, and adapted injury prevention training must already rank among the top priorities for future similar situations. This implies being creative, challenging, and using virtual platforms. One conclusion that can be drawn is the call for a slowdown. This could represent the opportunity to better protect athletes' health by taking sufficient time for a proper training programme including an exhaustive battery of tests. Finally, scheduling of competitions should be drastically adjusted; with the ultimate goal to put the athlete's health back at the core of the practice.

## Author Contributions

All authors listed have made a substantial, direct, and intellectual contribution to the work and approved it for publication.

## Funding

This work has been financially supported by the International Olympic Committee Medical and Scientific Commission Program for Prevention of Injury and Protection of Athlete Health.

## Conflict of Interest

The authors declare that the research was conducted in the absence of any commercial or financial relationships that could be construed as a potential conflict of interest.

## Publisher's Note

All claims expressed in this article are solely those of the authors and do not necessarily represent those of their affiliated organizations, or those of the publisher, the editors and the reviewers. Any product that may be evaluated in this article, or claim that may be made by its manufacturer, is not guaranteed or endorsed by the publisher.
